# Reviving degraded colors of yellow flowers in 17th century still life paintings with macro- and microscale chemical imaging

**DOI:** 10.1126/sciadv.abn6344

**Published:** 2022-06-08

**Authors:** Nouchka De Keyser, Fréderique Broers, Frederik Vanmeert, Steven De Meyer, Francesca Gabrieli, Erma Hermens, Geert Van der Snickt, Koen Janssens, Katrien Keune

**Affiliations:** 1 University of Antwerp, Department of Physics, AXIS Research Group, Groenenborgerlaan 171, B-2010 Antwerp, Belgium.; 2 Rijksmuseum, Museumstraat 1, Amsterdam, 1070 DN, Netherlands.; 3 University of Antwerp, Faculty of Design Sciences, ARCHES Research Group, Mutsaardstraat 31, B-2000 Antwerp, Belgium.; 4 University of Amsterdam, van ‘t Hoff Institute for Molecular Sciences, 1090GD Amsterdam, Netherlands.; 5 Utrecht University, Inorganic Chemistry and Catalysis, Universiteitsweg 99, 3584 CG Utrecht, Netherlands.; 6 Royal Institute for Cultural Heritage, Laboratories, Jubelpark 1, 1000 Brussels, Belgium.; 7 University of Amsterdam, Art History Department, Turfdraagsterpad 15-17, 1012XT Amsterdam, Netherlands.

## Abstract

Over time, artist pigments are prone to degradation, which can decrease the readability of the artwork or notably change the artist’s intention. In this article, the visual implication of secondary degradation products in a degraded yellow rose in a still life painting by A. Mignon is discussed as a case study. A multimodal combination of chemical and optical imaging techniques, including noninvasive macroscopic x-ray powder diffraction (MA-XRPD) and macroscopic x-ray fluorescence imaging, allowed us to gain a 3D understanding of the transformation of the original intended appearance of the rose into its current degraded state. MA-XRPD enabled us to precisely correlate in situ formed products with what is optically visible on the surface and demonstrated that the precipitated lead arsenates and arsenolite from the yellow pigment orpiment and the light-induced fading of an organic yellow lake irreversibly changed the artist’s intentional light-shadow modeling.

## Teaser

Combining chemical imaging methods enables reconstruction of the original appearance of a degraded yellow rose in a still life.

## INTRODUCTION

Over time, artist pigments and binders in oil paintings are inevitably subject to slow deterioration processes induced by external (light, relative humidity, temperature, and solvent exposure) and internal factors (copresence of incompatible pigment mixtures) (*1*, *2*). These complex processes take place at the paint surface or within the paint on the microscopic to the molecular level and can affect the physicochemical properties of the paint in an undesirable way. Inherent to the artist’s choice of material and technique, degradation entails discoloration and color changes and/or affects the structural integrity of the paint, causing (loss of) transparency, brittleness, delamination, or pronounced microcracking. At an advanced stage, these phenomena can decrease the readability of the artwork and hence conspicuously alter the artists’ intention. Notable examples of the degradation of artists pigments are the discoloration of the blue glass pigment smalt in Rembrandt paintings (*3*); ultramarine “sickness” (*4*) in works by Johannes Vermeer, Jan Steen, and Jan Van Eyck; the fading of light-sensitive pigments such as Prussian blues and organic yellow and red lake pigments; and the darkening of chrome yellows (*5*) and cadmium yellows (*6*) in paintings by, for instance, Edvard Munch, Vincent Van Gogh, James Ensor, Pablo Picasso, and other contemporaries. Because of pigment degradation, not only can carefully constructed optical effects such as folds in draperies disappear, rendering the object flat, for example, the ultramarine degradation in the blue cloak in Van Eyck’s *Three Marys at the Tomb* (1425–1435), but also a reverse optical effect can be formed [for example, *The Bedroom* (1888) by Van Gogh where the fading of the red pigments turned the purple walls blue and the pink floor brown (*7*)]. As paintings are composed of layers of complex heterogenous mixtures of pigments, it is often a combination of various degradation phenomena that affects the current appearance of degraded areas. Thus, to understand a degraded motif (e.g., draperies, flowers, foliage, incarnates, etc.) in itself and in context of the whole painting, a macroscale approach is required.

The capability of noninvasive imaging techniques such as macroscopic x-ray fluorescence (MA-XRF) scanning, reflectance imaging spectroscopy (RIS), and macroscopic reflectance Fourier transform infrared (MA-rFTIR) spectroscopy for the identification of artist’s working methods and materials has been demonstrated over the past decades (*8*–*10*). MA-XRF allows for the identification and mapping of chemical elements, while RIS provides complementary molecular information. A more recently developed method, macroscopic x-ray powder diffraction (MA-XRPD) imaging, has proven its potential in the cultural heritage field, specifically in the study of pigment degradation, as it allows for a noninvasive identification of crystalline pigment phases and secondary alteration products, as well as the visualization of their spatial distribution at the paint surface (*11*, *12*). When combined with microscopic imaging modalities [synchrotron radiation micro–x-ray powder diffraction (SR-μ-XRPD)], it becomes a highly valuable tool for obtaining a three-dimensional (3D) understanding of pigment degradation pathways (*13*).

In this context, arsenic-based pigments such as orpiment (As_2_S_3_) and realgar (As_4_S_4_), used by artists since antiquity, are known to undergo several discoloration phenomena and have severely affected the current appearance of various old master paintings. Paolo Veronese, Tintoretto, and Giorgione used the arsenic pigments in orange draperies, while Dutch and Flemish still life painters such as Adriaen Coorte, Daniël Seghers, Willem Kalf, and Jan Davidsz. De Heem made use of their ideal paint properties in oil to create luminous golden or warm glowing highlights to paint oranges, lemons, yellow flowers, or a sheen of golden metalwork (*14*–*18*). Despite numerous documentary sources with disclaimers warning against their incompatibility with other pigments, poisonous nature, horrible drying property, and change of color, they were still used until the 18th century and now are often found degraded (*19*). Earlier studies have pointed out the light sensitivity of these pigments through photo-oxidation with the formation of secondary products such as white arsenolite (As_2_O_3_) (in case of the yellow pigment orpiment) and bright yellow pararealgar (As_4_S_4_) (in case of the orange pigment realgar) (*15*, *20*). Keune *et al.* (*14*) found that highly mobile and soluble arsenate (As^5+^) species, as a result of degradation, migrate through the multilayered paint system. Recently, two rare lead arsenate species, mimetite [Pb_5_(AsO_4_)_3_Cl] and schultenite (PbHAsO_4_), were identified in still life paintings by Jan Davidsz. De Heem and Martinus Nellius and are considered to be degradation (end) products of those soluble mobile arsenates that have precipitated with available Pb^2+^ ions (*13*, *21*). While these studies dealt with the characterization of the arsenic species, this work demonstrates how the multimodal combination of chemical and optical imaging techniques allows us to understand and document the visual impact of arsenic degradation on the optical appearance of the paint and the painting itself with remarkable detail.

**Fig. 1.**
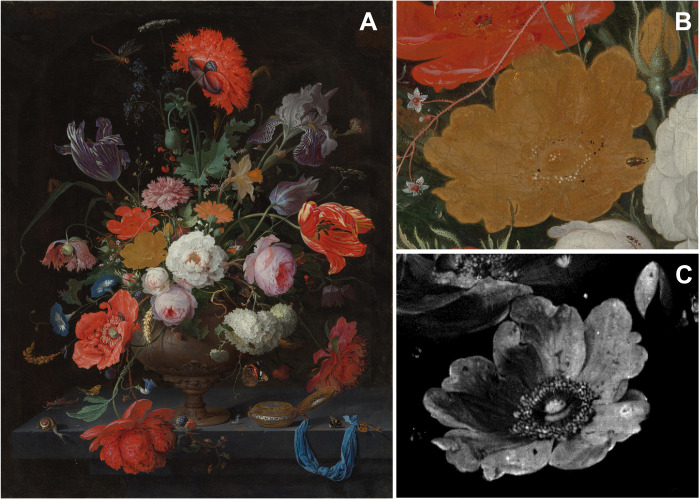
Pigment degradation in the yellow rose of Abraham Mignon’s *Still Life with Flowers and a Watch.* (**A**) Visual photograph of *Still Life with Flowers and a Watch* of Abraham Mignon (1640–1679), oil on canvas, dated c. 1660–1679, signed *A. Mignon. Fc*, from the collection of the Rijksmuseum (SK-A-268), (**B**) with a detail of the yellow rose (**C**) and the corresponding elemental distribution image of arsenic.

As a case study for this research, we focus on the yellow rose in the painting *Still Life with Flowers and a Watch* by Abraham Mignon (1640–1679) from the collection of the Rijksmuseum. Since long, yellow roses have attracted the attention of scholars, as these are considered signature flowers in the circle of the leading 17th century still life painters Jan Davidsz. De Heem (1606–1684), Abraham Mignon, and Daniël Seghers (1590–1661). In addition, several authors identified the presence of natural orpiment as their main constituent pigment, whereas yellow roses have been reported to exhibit varying degrees of degradation (*22*–*25*). Mignon’s yellow rose in the Rijksmuseum painting is therefore an exemplary topic for this study, looking flat and poor in color contrast while featuring a crumbling powdery appearance or a conspicuously broken up paint surface. In particular, upon considering the surrounding, better-preserved flowers in the composition, it becomes clear that the rose has lost most of its 3D character (*22*, *23*). Nevertheless, the elemental map of arsenic, supplied by MA-XRF imaging experiments, revealed that the paint layers still hold all details of the once elaborated brushwork used to create the 3D illusion of the flower that is now no longer visible to the naked eye (Fig. 1).

The question of why we cannot perceive these details anymore can be raised. This research therefore aims to gain an in-depth 3D understanding of the transformation of the original intended appearance of the yellow rose into its degraded current state by (i) identifying the original pigments and their stratigraphy, to gain a complete understanding of the paint technique, and (ii) characterizing the secondary degradation products on the micro- and macroscale, to better understand the various degradation processes that have occurred. To do this, the painting was analyzed with a range of analytical imaging techniques, which includes high-resolution elemental mapping of the yellow rose with the Bruker M6 MA-XRF scanner, MA-XRPD imaging in reflection mode (fig. S1) with x-rays probing superficial paint layers under a shallow angle (10°), and RIS in the visible to near IR (VNIR). To visually correlate the results of the chemical imaging techniques, the yellow rose paint surface was photographed with a 5-μm pixel resolution and studied under the 3D Hirox surface microscope for midrange resolution images. Complementary to the noninvasive analyses, a microsample taken from the yellow rose was examined with scanning electron microscopy combined with energy-dispersive x-ray (SEM-EDX) analysis and with SR-based x-ray powder diffraction at the P06 beamline at PETRA III (DESY). Additional characterization of the original arsenic-based pigment was done using micro–Raman spectroscopy.

## RESULTS

### Noninvasive analysis: MA-XRF

**Fig. 2.**
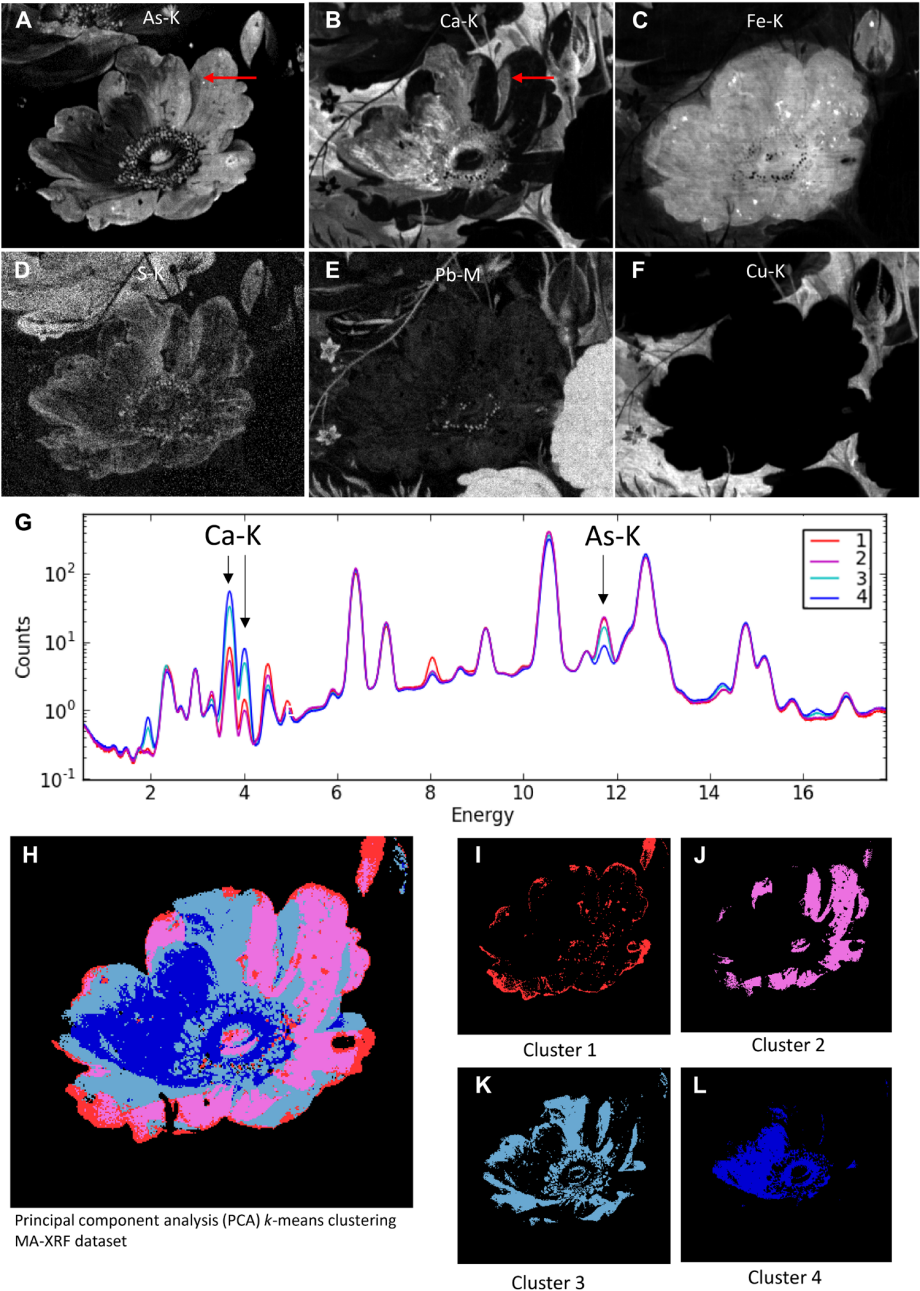
Results from MA-XRF imaging and principal component analysis with *k*-means clustering of the dataset. Detailed elemental distribution images for (**A**) arsenic, (**B**) calcium, (**C**) iron, (**D**) sulfur, (**E**) lead, and (**F**) copper of the yellow rose acquired with a Bruker M6 Jetstream by mapping an area of 193 mm by 235 mm with a step size of 300 μm and a dwell time per pixel of 150 ms. Results from the principal components analysis (PCA) with *k*-means clustering showing the averaged XRF spectra of the clusters 1 to 4 in (**G**) and the clusters visualized in a composite image (**H**) and separately (**I** to **L**).

MA-XRF imaging enabled mapping the distribution of the elements present in the yellow rose; relevant distribution maps are shown in Fig. 2: for arsenic (A), calcium (B), iron (C), sulfur (D), lead (E), and copper (F). The arsenic, calcium, and sulfur maps demonstrate painterly features for the definition of the flower, while in the iron map, the overall shape of the flower can be discerned. Following Mignon’s use of light and shadow in surrounding flowers, the yellow rose would have been illuminated from the upper left. Although the contrast, light, and shadow modeling is no longer visible today, the elemental distribution maps of arsenic and calcium give insight into the original illumination. While the arsenic map visualizes the light striking the flower with intricate details and highlights that were meticulously applied to define the flower petals and stamens, the distribution of calcium appears to correlate with the expected shadow areas. This is particularly visible where one of the upper flower petals casts a shadow on a neighboring petal [see red arrows in Fig. 2 (A and B)].

In the distribution map of iron, both the rough shape of the flower and an oval shape encompassing the flower are visible. The iron signal does not correspond to any painterly feature in the flower and neither correlates with the arsenic or the calcium map; this suggests that the signal is stemming from a more uniformly applied underpainting underneath the flower. From previous MA-XRF studies on Dutch flower still lives, a single oval-shaped underpainting would be expected here, marking the position of the flower applied in an earlier stage. The iron signal from the lower underpainted layer is attenuated differently by the superimposed layers (*18*, *25*, *26*). The background around the flower is applied with a copper-rich paint (Fig. 2F) that is blocking the iron signal of the underpaint more than the superimposed calcium and arsenic paint layers constituting the flower. Little white highlights are also present in the center of the flower and were applied with touches of lead white, visible in the lead distribution map (Fig. 2E).

A closer inspection of the MA-XRF dataset was obtained by performing principal component analysis (PCA) followed by *k*-means clustering in PC space. The first six PCs covered most of the variance in the data and were used for image segmentation by *k*-means clustering. Pixels with similar spectra are effectively clustered via this method into *k* groups, resulting in an image segmentation. To achieve additional image segmentation, 13 clusters were selected (without prior knowledge), and the result was improved by a Gaussian mixture model (GMM) using expectation maximization (EM) for clustering (maximum of 1000 iterations) (fig. S2). In Fig 2, the composite image (Fig. 2H) and four separate clusters (Fig. 2, I to L) that describe the different pixel types within the yellow rose are presented. The average spectra of the four clusters are reported in Fig. 2G; the largest difference between the four clusters can be explained by the variance in observed arsenic and calcium XRF intensity.

**Fig. 3.**
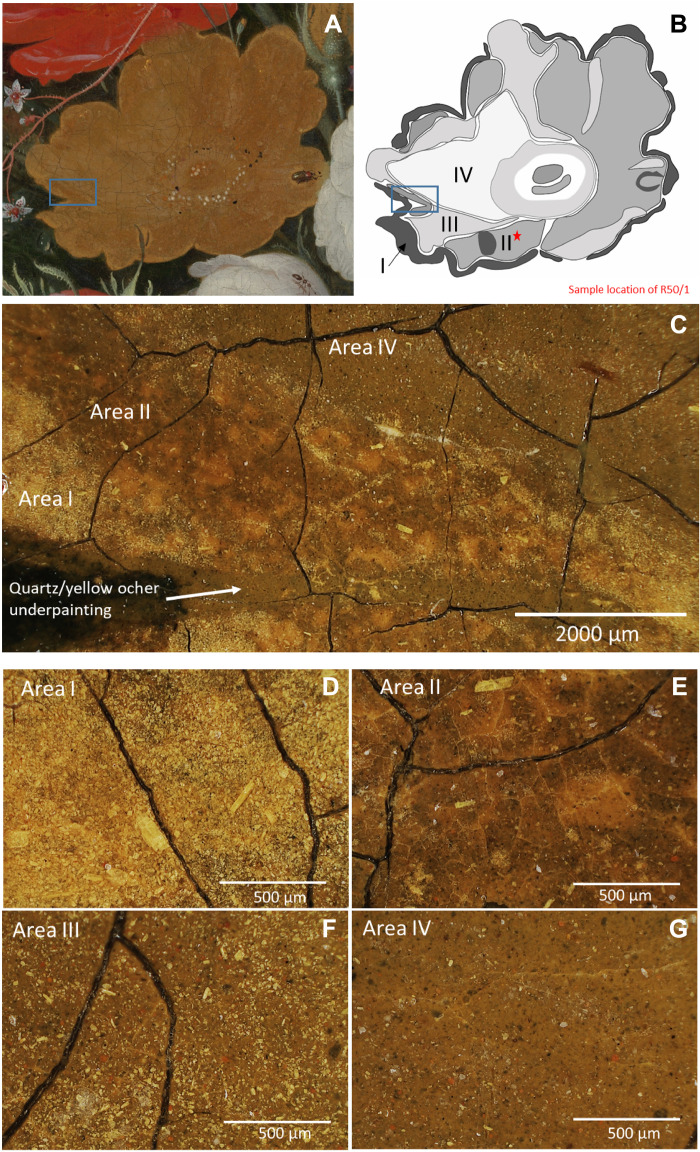
Comparison of the differences in optical appearance in the yellow rose under the 3D digital optical microscope. (**A**) Detail of the yellow rose. (**B**) Schematic overview of the four defined areas (I to IV) and sample location, (**C**) detail of the rose photographed under low magnification (×35), and corresponding images photographed under the Hirox microscope with magnification of ×140 for area I (**D**), area II (**E**), area III (**F**), and area IV (**G**).

When visualizing the location of these clusters in the composite image (Fig. 2H), it becomes apparent that the PCA with *k*-means clustering effectively identified pixel groups within the yellow rose that also, under the 3D digital optical microscope, can visually be differentiated on the basis of their tonality, paint surface, particle distribution, and condition. While from a distance, subtle nuances, details, and transitions can hardly be observed in the yellow rose, up close and under the microscope, the differences in optical appearance among the four areas is evident. In Fig. 3B, the areas are indicated on the yellow rose and supplemented with microscopic images taken at medium magnification (×140) under the 3D digital optical microscope (Fig. 3, D to G).

Area I, closely corresponding to cluster 1, defines the more bright yellow color around the outline of the yellow rose and the highlight under the little beetle and leaf. Under the microscope, this area (Fig. 3C) is specifically characterized by a clustering of bright yellow pigment particles with a foliated micaceous platelet-like morphology, similar to natural orpiment. The size of the particles (lengthwise) varies from small (c. 20 μm) to coarse (on average, 100 to 200 μm up to even 600 μm for a few particles).

The paint surface indicated as area II (Fig. 3D), correlating with cluster 2, represents most of the paint surface and shows clear signs of degradation. It has a brittle appearance due to a more pronounced microcracking and is affected by superficial paint losses. Visually, the area appears darker in tonality, and under the 3D digital optical microscope, it is perceived as a darkened more transparent paint with fewer bright yellow particles (ranging from 20 to 100 μm).

By eye, the transition between area II and area III in the painting is subtle; however, area III (Fig. 3E), closely corresponding to cluster 3 (Fig. 2K), is lighter gray in tonality, appears less brittle, and is not affected by paint loss. Under the 3D digital microscope, again, the same bright yellow pigment particles are observed but in greater quantity and more evenly distributed over the surface in a transparent layer compared to the sporadically distributed particles observed in area II.

Area IV indicates a region located on the left side of the rose (Fig. 3F) [correlating with cluster 4 (Fig. 2L)] and is visually perceived whiter in tonality with a powdery appearance compared to the neighboring areas. Under the 3D digital optical microscope, mostly white particles and few yellow particles can be observed in an overall transparent-looking paint film over a coarse-grained underlayer.

Although MA-XRF scanning provides highly specific information on the macroscale, it is not depth selective and elemental information can originate from different levels in the stratigraphy, which has to be taken into account when interpreting the resulted clustering. There is a variable degree of attenuation of the signal, depending on the thickness, atomic number, and density of superimposed materials. For instance, the area on the top where the yellow flower petal was painted over the neighboring red flower erroneously resulted in a different clustering because of attenuation of the iron signal (present in the underpainting) by the superimposed vermilion (HgS) paint of the red flower (fig. S2). Although the obtained clusters merely group pixels with similar spectra, rather than showing exact paint mixtures that were used to paint the flower, in this case, the clusters could overall be linked with visually distinct areas of the paint surface and even prelude to the presence of different calcium-based pigments and formed degradation products, which will be discussed in the next paragraph.

### Noninvasive analysis: MA-XRPD

**Fig. 4.**
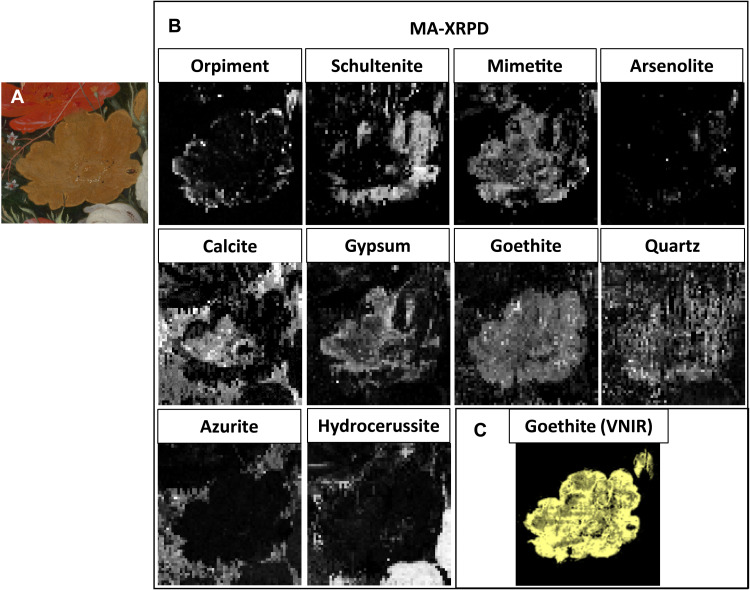
Results from MA-XRPD in reflection mode and VNIR. (**A**) Optical photograph of the analyzed area. (**B**) Distribution images obtained with reflection MA-XRPD, showing the intensity scaling parameter for every pixel. (**C**) Map obtained with the portion of the spectral endmember showing characteristic features of goethite, contained in the yellow ocher pigment.

To better distinguish the pigments present in the superficial paint layers and identify the formed degradation products (i.e., the different arsenic and calcium phases), MA-XRPD maps of the yellow rose were recorded. The painting was analyzed in reflection mode with monochromatic x-rays impinging the paint surface under a shallow angle (10°), which limits the probing depth to the superficial paint layers of the stratigraphy, ranging between 10 and 50 μm, depending on the material composition (*21*, *27*). An area of 97.5 mm by 109.5 mm was scanned with an exposure time of 10 s per pixel and a step size of 1.5 mm by 1.5 mm. In Fig. 4, a selection of the relevant MA-XRPD distribution maps is shown. These distributions allowed further interpretation of the aforementioned MA-XRF results (Fig. 2) and microscopic observations of the paint surface (Fig. 3), i.e., by enabling a correlation between specific crystalline pigment phases and degradation products with the MA-XRF clusters and optical areas, respectively.

The elemental arsenic maps were substantially augmented by MA-XRPD, as no less than four different arsenic-containing crystalline products were identified: schultenite (PbHAsO_4_), mimetite [Pb_5_(AsO_4_)_3_Cl], arsenolite (As_2_O_3_), and orpiment (As_2_S_3_) (fig. S3), each showing a distinct spatial distribution. In particular, orpiment, schultenite, and mimetite correspond to areas I, II, and III, respectively, while arsenolite can be visually correlated to the white crust formations in area II. In addition, two main calcium-based crystalline products: calcite (CaCO_3_), and gypsum (CaSO_4_·2H_2_O) were found, with calcite corresponding to the calcium rich area IV, while gypsum matches area III with a similar distribution as mimetite. Last, goethite [FeO(OH)] and quartz (SiO_2_) are present.

Both mimetite and schultenite are rare arsenate minerals in nature and, consistent with recent literature, can be identified as secondary degradation products in artworks (*13*, *15*, *21*). Their different spatial distribution in the rose is particularly of interest, as it seems that the distribution of mimetite is correlated to the distribution of gypsum. Local environmental conditions play an important role in the in situ formation of crystalline products in paint layers; the conditions for the formation of mimetite or schultenite depend on several factors. In general, As(V) species, originating from an oxidation reaction of As(III) in orpiment, are predominant under moderate to high oxidation conditions, in this case, a more oxidized paint medium (*28*). Mimetite is relatively insoluble and thermodynamically stable, particularly at pH > 5 (*22*), while schultenite is formed under more acidic conditions. In addition, mimetite also requires the presence of chlorine (Cl^−^ ions), which are often present in old master paintings and can stem from the use of the Dutch stack process for the manufacture of lead white, can be released during lead soap formation, or can originate from external (atmospheric) sources (*13*).

The more abundant presence of gypsum and calcite in area III might be indicative of a more alkaline environment, favorable for the formation of mimetite. Furthermore, we could hypothesize that such a variation in pH could have been caused by a variability in the local concentration of orpiment in the paint matrix, with more orpiment initially present in area II than in area III.

Although MA-XRF scanning is less sensitive to elements with a low atomic number (*29*), the elemental distribution of sulfur is consistent with the above-mentioned MA-XRPD results, in particular, showing less S to be present in area II, rich in schultenite, than in the orpiment-rich border of the flower (area I), while the arsenic distribution map shows a comparable arsenic content in areas I and II.

In addition, MA-XRPD mapping provided information regarding the iron-containing pigment in the presumed underlying layer and the distribution of quartz over the whole surface of the flower. In contrast to the elemental map of iron obtained by MA-XRF, the map of goethite, indicative of yellow ocher, shows only the shape of the flower and not the oval shape surrounding it. The copper (azurite containing) background paint covering the ocher blocks the goethite signals from the underlying oval shape.

Complementary to MA-XRPD, RIS in the VNIR spectral region allowed us to map goethite within the painting, giving rise to comparable distribution maps (Fig. 4C). RIS in the visible spectral range detects spectral signatures from pigments that are predominantly present on the surface of the painting based on the electronic transitions related to the color of materials (*30*). The VNIR goethite map was obtained on the basis of a spectral endmember showing the typical features for goethite: a reflectance transition edge around 545 nm and absorption bands near 650 and 950 nm (fig. S4) (*30*). Yellow pigments such as orpiment and nondegraded yellow lake pigments normally can be detected with VNIR; however, in this case, no definite spectral features for their identification could be observed (*31*). We attribute this to the fact that, as demonstrated by MA-XRPD analysis, the original yellow pigment at the surface has converted into secondary products and that the yellow lakes faded.

### Microscale analysis

A cross-sectioned sample (R50/1), taken from area II of the yellow rose, offered the opportunity to study the buildup, pigment composition, and distribution of secondary degradation products on the microscale. The cross section was studied under the light microscope, and elemental and molecular information was obtained by SEM-EDX analysis, Raman spectroscopy, and transmission mode SR-μ-XRF/XRPD mapping performed at beamline P06 of the PETRA III facility to provide complementary information to the macroscale XRF and XRPD analyses.

**Fig. 5.**
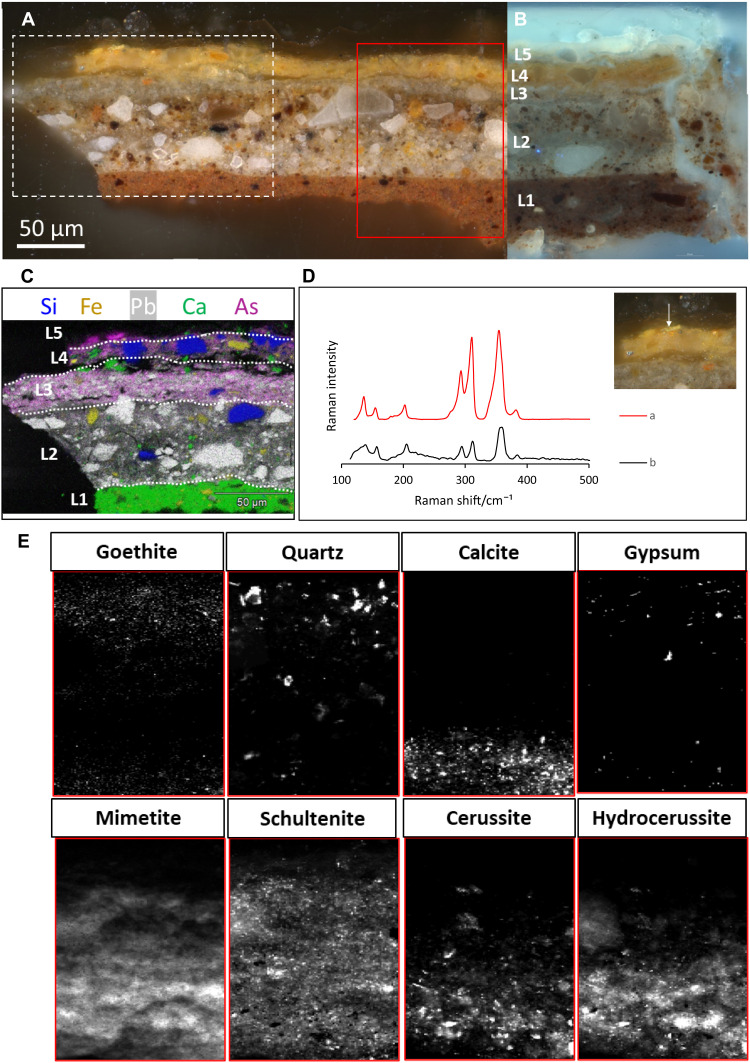
Results of microscale analysis [light microscopy (LM), SEM-EDX, Raman, and SR-μ-XRPD] of the paint cross section taken from the degraded yellow rose (sample location is indicated in Fig. 3B with a red star). (**A**) Cross section R50/1 photographed in dark field (A) and UV light (**B**). The white rectangle indicates the area analyzed by SEM-EDX, with ensuing elemental maps shown in (**C**) of silicon (Si), iron (Fe), lead (Pb), calcium (Ca), and arsenic (As). The red rectangle indicates the area imaged by SR-μ-XRPD, with ensuing results shown in (E). (**D**) Identification of natural orpiment of an arsenic sulfide particle (white arrow) in cross section R50/1 with Raman spectroscopy: (a) reference spectrum of natural orpiment (RRUFF ID: R060105) and (b) spectrum collected on an orpiment pigment particle in R50/1. (**E**) Crystalline pigment phases and secondary products obtained with SR-μ-XRPD mapping at the P06 beamline of PETRA III. The 2D distributions shown are based on the scaling factors obtained through whole-pattern fitting, and the collected diffraction data were corrected for attenuation effects.

In Fig. 5E, the distribution maps of the most relevant crystalline phases are shown; five paint layers could be identified. Unfortunately, since the layered structure of the sample did not align completely to the incoming x-ray beam (because of a slight tilt of the sample in the embedding material), the boundaries between the different strata cannot be easily discerned. However, combined with the corresponding XRF maps (obtained simultaneously during the μ-XRPD analysis) and with the SEM-EDX point and mapping analysis (Fig. 5C), it was nevertheless possible to obtain a full understanding of the (original) sample stratigraphy: a red brownish calcite ground layer (L1) followed by two lead white–containing layers (L2 and L3), a yellow goethite + quartz layer (L4), and a thin, transparent top layer (L5) with one remaining orpiment particle.

The first three layers (L1 to L3) of the cross section are consistently found in other cross sections taken from this still life painting and are consequently not specific for the buildup of the yellow rose. Mignon made use of a gray (L3) on double ground (L1 + 2) as traditionally encountered in 17th century Dutch paintings on canvas (*32*). The first red ground (L1) is mainly chalk based (calcite) and consists of earth pigments such as hematite, goethite, and lead white. The second grayish-brown ground (L2), which is approximately 50 μm, is mainly lead white based (cerussite and hydrocerussite) and contains quartz and earth pigments. The third, thin whitish gray layer (L3) consists of primarily lead white (cerussite and hydrocerussite), chalk (calcite), and fine carbon black particles. Layer 4 is a warm yellow paint layer that is approximately 20 μm thick and appears to be mainly composed of coarse quartz (SiO_2_) particles, fine-grained goethite, and some lead white, as identified by SR-μ-XPRD and EDX mapping and point analysis. In view of its goethite content, L4 is associated with the iron-rich underpainting layer that was previously inferred from macroscale imaging. Microanalysis on the paint cross section now evidences that the goethite layer is only followed by a thin (semi-)transparent paint layer (L5) in the paint stratigraphy, which aided its detection by the superficial MA-XRPD and RIS techniques. Layer 5 appears very thin and transparent in bright-field light microscopy but is highly fluorescent under ultraviolet (UV) light (Fig. 5B). The elemental SEM-EDX images and, in particular, additional point analyses revealed a pigmented layer containing quartz, calcite, gypsum, and one arsenic sulfide particle (the only yellow pigment particle present at the far left in layer 5). Both the refractive index of calcite, gypsum, and quartz are close to the binding medium of oil, which partially explains the transparent aspect of this paint layer. Today, the aforementioned Hirox observations exposed the top paint layer (L5) in area II as a darkened, semitransparent, and brittle paint over a coarse-grained brown underlying layer, corresponding to layer 4 in the paint cross section.

Although arsenic seems to be present throughout the paint stack, both SR-μ-XRF mapping and SEM-EDX analysis found only one remaining, intact orpiment grain, i.e., in L5. We therefore hypothesize that the artist included orpiment exclusively in the top paint layer. Micro–Raman spectroscopy identified this grain as natural orpiment, as demonstrated by the characteristic bands of natural orpiment with very strong absorption peaks at 311 and 355 cm^−1^, strong peaks at 293 cm^−1^, medium peaks at 154 and 203 cm^−1^, and weak to very weak peaks at 136 and 106 cm^−1^ (Fig. 5D) (*33*). Supplementary to MA-XRPD, SR-μ-XRPD imaging did not only allow for the identification of the secondary arsenic products schultenite and mimetite but also permitted to situate their distribution over the layer buildup (fig. S5). Both lead arsenates are not located in discrete layers but are found distributed throughout the paint stratigraphy from L2 to L5, in this way, explaining the aforementioned elemental distribution. The ample presence of schultenite and mimetite throughout the stratigraphy is considered to be the result of a multistep degradation process of arsenic sulfide pigments, with soluble arsenate species migrating from L5 through the paint stratigraphy and locally precipitating into lead arsenate minerals upon encountering suitable Pb^2+^ ions from (saponified) lead carbonate (*13*). Preparatory layers L2 and L3 are particularly rich in hydrocerussite and cerussite and thus provide a good lead source for the formation of lead arsenates. The migratory aspect is underpinned by the backscattered electron image (fig. S6), revealing the presence of sharp needle-like particles, rich in arsenic and lead, and visible throughout layers 2 to 4 (*14*).

## DISCUSSION

### Painting technique

On the basis of the chemical imaging on the micro- and macroscale and the optical examination of the paint surface, the original painting technique and layer buildup of the yellow rose could be inferred. The flower appears to have been painted after an efficient three-step method that is characteristic for 17th century still life painters. This included first blocking in the position of the flower with a monochrome underpainting and subsequently working up the details by applying (semi)transparent paint, i.e., with glazes for the shadows and lighter scumbles of paint for the sunlit parts (*24*). In this manner, Mignon marked the position of the yellow rose with a yellow ocher-based underpaint that provided a midtone base for the superimposed top paint layer. The main body and details of the flower were painted with different orpiment containing paints depending on the desired/intended hue: either exclusively pigmented with orpiment for the bright highlights in area I or with more increasing admixture of gypsum, calcite, quartz, and carbon black for the light midtone of area II and the intermediate shadow tone of area III. In the expected deeper shadows, a more abundant presence of calcite was detected (area IV), which suggest the presence of a yellow lake.

A direct parallel can be drawn from Mignon’s painting technique with the contemporary Dutch painters manual *The Big World Painted Small* by Willem Beurs, published in 1692 in Amsterdam. Aside from other detailed instructions for various objects in still life paintings, he includes paint instructions for the yellow rose [*geelen eglantier*] and suggests to paint it only with Kings yellow [*koningsgeel*], the artificial equivalent of natural orpiment, for the highlights; a little bit of carbon black and a fugitive yellow lake [*schietgeel*] for the shadows; for the reflections, only a light yellow lake; and for the colors in between, a mixture of Kings yellow and black.

In Mignon’s yellow rose, calcite is present in the areas that we assume to have been shadowed, and although it is plausible that chalk was used as a filler for the translucent glaze, it is more likely present as a residual substrate for a now degraded yellow dyestuff, as its use is also prescribed by Beurs. Lake pigments were prepared by the precipitation or adsorption of an organic dyestuff onto an insoluble substrate. For yellow lakes in 17th century paintings, calcium carbonate or chalk is almost predominantly found as a substrate (*34*–*36*). The presence of a yellow lake is generally difficult to prove, as the yellow organic dyestuff has completely faded away as a result of photochemical reaction. However, previous studies have demonstrated that its presence can be indicated by a high concentration of calcium (as a marker for chalk) (*37*). Chalk in its own right was sometimes used as a paint additive, to give body and translucency to a paint, but typically in combination with other colored pigments. This does not appear to be the case here, given that no other elements or colored pigments seem to correlate with area IV on the basis of the MA-XRF and MA-XRPD results. Thus, this strengthens our hypothesis regarding the use of a degraded yellow lake with a Ca-based substrate.

In area III, a clear amount of gypsum is found, associated to the paint mixtures with orpiment and little calcite. It has been suggested that gypsum can be formed in situ as a secondary product, as a reaction between calcite and orpiment in the oxidized paint matrix. However, this would entail the presence of smaller (nano-like) particles formed through precipitation, while in this case, bigger crystal particles (2 to 10 μm) are observed inside the paint, which suggest the presence of deliberately added gypsum. Gypsum does not have a considerable color effect in oil, and from paint reconstructions made by the author with natural orpiment, the addition of gypsum also does not influence the color of the bright yellow orpiment. It does however change the optical properties to a more translucent yellow paint, which, applied on a quartz and yellow ocher paint, gives a subtle warm greenish hue. This paint layer thus likely functioned as a pigmented glaze to achieve an intermediate shadow tone required for the smooth transition between area II and area IV. Gypsum is listed by the Italian painter Lomazzo in his *Trattato dell’arte de la pintura* among the pigments that are safe to use with orpiment, and the combined detection with gypsum has also been reported in other paintings by Adriaen Coorte, Jan Davidsz. de Heem, and Martinus Nellius(*13*, *15*, *38*, *39*).

Quartz (SiO_2_) was identified with SR-μ-XRPD in L4 and L5 of the paint cross section, whereas MA-XRPD found it distributed over the entire surface of the flower (Fig. 4). Quartz is a mineral commonly found in iron and arsenic ores. However, in view of the size of the particles and their abundance, quartz seems to also have been intentionally added to the paint mixture. Under the SEM microscope, the particles show a distinct angular shape characteristic of crushed glass, but EDX spectra of individual particles only presented high signals for silicon and oxygen, excluding the hypothesis of the presence of colorless glass, in which considerable amounts of sodium, potassium, and calcium or minor quantities of magnesium, aluminum, phosphorus, titanium, manganese, or iron are expected (*40*). Orpiment is typified in historical sources as a poor drier and an extremely hard pigment to grind. Authors such as Cennino Cennini (*41*), Marshall Smith (*42*), Palomino (*43*), De Mayerne (*44*), and Willem Beurs (*45*) therefore recommend the addition of glass to improve its drying and grinding properties (*46*). Although more inert than glass, paint reconstructions and grinding experiments proved that quartz also facilitated the grinding and the drying of the orpiment paint. Another plausible explanation for the presence of quartz in the paint mixtures is that it may have functioned as a transparent, colorless extender to improve the working properties of the paint and/or to change the physico-optical properties of the paint film, increasing its transparency (*46*). Quartz or other silica are regularly encountered in paint, but its function is rarely discussed and not yet well understood, for example, in the grounds of Rembrandt paintings (*47*) and mixed with a red lake in Lorenzo Lotto’s *St. Catherine*, dated 1522 (*46*).

### Optical changes

The combined analysis augmented with close examination of the paint surface led to important insights on the now degraded state of the yellow rose. As a result of various chemical and physical degradation processes in the paint system, the originally intended optical appearance of the yellow rose drastically changed. The yellow lake, used to paint the shadow areas on the flower, faded, while the orpiment, used in the sunlit area of the flower, degraded and turned the paint layer from bright yellow to transparent. The degradation of the original parent pigment instigated various reactions, which led to the formation of arsenolite, schultenite, and mimetite. Intact orpiment particles are still present around the borders of the yellow rose (area I) and dispersed over the surface of the yellow rose. These are probably the paint areas with the highest concentrations of orpiment, leaving some grains only partially degraded. Arsenolite can be visually correlated to white crust formations on the paint surface as a result of direct photo-oxidation of the orpiment, while the increased transparency and consequent visual darkening in area II and particularly in area III are caused by the physical breakup of the yellow mineral and subsequent formation of the colorless secondary lead arsenate minerals schultenite and mimetite. The degradation now allows light to penetrate the once opaque top paint layer and be reflected by the underlying dark yellow ocher-based underpainting, leading to an optical darkening effect.

Both pigment mixtures that were used for creating either the shadows on the flower or the bright yellow highlights degraded or faded, and while these paint layers were intentionally already thinly applied, conforming to the painting technique of still life painters, both have caused an increased visibility of the underlying, monochrome yellow ocher paint layer, which is now responsible for the overall color appearance of the rose. This resulted in a flatter (less 3D)–looking flower, as subtle transitions defining the body of the flower can no longer be perceived, which is the reverse optical effect originally intended by Mignon.

In conclusion, a combined micro- and macroscopic approach has been presented in this article to investigate the yellow rose in *Still Life with Flowers and a Watch* by Abraham Mignon. This investigation has led to a considerably improved insight into the bilayer buildup, current condition, and the original intended appearance of this strongly degraded flower.

Degradation of the arsenic pigment orpiment and yellow lake has each caused substantial optical changes. Light-induced fading of the organic yellow dyestuff in the yellow lake glaze has caused the intended shadow areas to turn white because of the remaining calcite-based substrate. The yellow bright pigment orpiment underwent several chemical reactions, leading to the precipitation of lead arsenates schultenite and mimetite and the formation of arsenolite. These newly formed degradation products visually affected the paint surface. White crust deposits on the paint surface were correlated to arsenolite, while the formation of schultenite and mimetite altered the physical condition and appearance of the paint. The once bright yellow paint transformed into a colorless, transparent, and brittle layer. Although the chemical reactions irreversibly changed the artists’ intention, chemical imaging such as MA-XRF scanning made it still possible to recapture the once visible and meticulously applied highlights and shadows of this flower.

From a wider perspective, this paper showcases the potential of SR-μ-XRPD and MA-XRPD to discriminate inorganic degradation products at the micro- and macroscale. MA-XRPD enables us to precisely correlate in situ formed products with what we see on the surface, while SR-μ-XRPD investigations made an in-depth study of this particular degraded multilayered system possible on the microscale. RIS in the VNIR spectral range also provided insight into the optical condition of the now-degraded layers, as their consequent transparency allowed mapping goethite in the underpainting with high spatial resolution.

Ultimately, this interdisciplinary approach uncovered new information on Mignon’s artistic practice, and in a broader perspective, our research sheds light on substantial optical changes that might occur for specific surface textures that were painted with a similar pigment palette. Our methodology and all the extracted data that we obtained toward a virtual reconstruction of a degraded motif could be applied to artworks of worldwide renown.

## MATERIALS AND METHODS

### Microscale analysis

#### Light microscopy

A microscopic paint sample (R50/1), taken by A. Wallert in 1999 (*24*), was reexamined for the purpose of this study. The cross section was taken from a flower petal of the yellow rose (see figure for the location of the cross section). The cross section was photographed according to the paint sample database protocol of the Rijksmuseum with a Zeiss Axio Imager.A2m microscope (Carl Zeiss Microscopy LLC, USA) equipped with a Zeiss AxioCam Mrc5 digital camera. White light was provided by a light-emitting diode (LED) lamp with a color temperature of 5600 K and a colibri.2 controller for UV fluorescence microscopy (LED, 365 nm). All images were obtained, processed in the image acquisition software Zen 2 pro (blue edition) with extended depth of focus facilities, and observed on a calibrated EIZO ColorEdge CG277 BK computer screen.

#### Scanning electron microscopy energy-dispersive x-ray spectroscopy

SEM-EDX was performed on a JEOL JSM-5910LV microscope. Before the analysis, the cross section was coated with a thin layer of gold to improve surface conductivity using a JEOL JFC-1200 Fine Coater (15 s at 45 mA). Backscattered electron images were obtained in high vacuum and point analysis and elemental mapping using the Noran System Six software. The cross section was also studied in low vacuum with the FEI scanning electron microscope. Backscattered electron (BSE) images of the cross section were taken at an acceleration voltage of 5 kV, at a 5.9-mm eucentric working distance with a spot size of 3.

#### SR-μ-XRPD

To gain more insight into the crystalline pigment phases and secondary degradation products, μ-XRPD mapping was carried out on cross section (R50/1) at the Microprobe hutch of the hard x-ray micro/nanoprobe beamline (P06) of the PETRA III storage ring (DESY, Hamburg, Germany) (*48*). A Kirkpatrick-Baez mirror optical system was used to focus the beam, achieving a size of 0.5 μm by 0.5 μm (hor. × vert.), using a primary photon energy of 21 keV. A Keyence optical microscope equipped with a perforated mirror was allowed for positioning of the sample. Diffraction signals were recorded in transmission geometry using an EIGER X 4M area detector (Dectris Ltd., Switzerland), and calibration of the diffraction setup was performed by means of a LaB_6_ reference sample.

An area of 100 μm by 180 μm was scanned using a step size of 1 μm in the horizontal direction and 0.5 μm in the vertical direction. An exposure time of 0.25 s per pixel was used to acquire the diffraction patterns.

The in-house developed software package XRDUA was used for the processing of the XRPD data. The obtained 2D distributions shown in this article are based on the scaling factor obtained through whole pattern fitting, and the collected diffraction data were corrected for attenuation effects (*49*).

#### Micro–Raman spectroscopy

Micro–Raman spectroscopy spectra were acquired with a Renishaw InVia Raman microscope with a Peltier-cooled (−60°) charge-coupled device detector (1020 × 256 pixels), using a high-power near-IR solid diode laser of 785 nm (1.12 mW) in combination with a grating of 1200 lines per millimeter (l/mm). A silicon reference sample was used to calibrate the instrument. To avoid damage, the power of the laser was reduced to 0.1% with neutral density filters. The sample was analyzed with a ×100 objective and an exposure time of 2 s and five accumulations. The spectra were acquired, normalized, and corrected for baseline using the Wire 3.4 Raman software.

### Macroscale analysis

#### MA-XRF imaging

Elemental maps of the entire painting and a smaller detail were collected with the commercially available MA-XRF scanner M6 Jetstream from Bruker Nano GmbH (Berlin, Germany). The M6 Jetstream consists of a 30-W Rh-target microfocus x-ray tube with a maximum voltage of 50 kV, a maximum current of 0.6 mA, a polycapillary lens, and a 30-mm^2^ X-Flash silicon drift detector that is moved over the surface of the painting by means of an X, Y–motorized stage, enabling a scan area of 80 cm by 60 cm (*50*). A spot size of 150 μm was set for the measurements with a working distance of ca. 10 mm from the x-ray snout and the surface of the painting. The entire painting (750 mm by 571 mm) was scanned with a step size of 650 μm and a dwell time 70 ms. For the detail scan (193 mm by 235 mm), including the yellow rose, a step size of 300 μm and a dwell time of 150 ms were used.

The resulting spectral data cubes of the MA-XRF scanning were processed using data analysis software packages Python Multichannel Analyzer and Datamuncher gamma (*51*, *52*). In the ensuing 2D distribution maps, each pixel carries information on the calculated net peak intensities of the emission lines of the element, with a gray scale linear to the detected intensities.

### PCA: *k*-means clustering

For a closer inspection of the MA-XRF dataset, a PCA with subsequent *k*-means clustering was carried out using the open-source software package XANES-wizard.(*53*, *54*). PCA is a well-established dimension-reducing statistical technique, applied for pattern recognition in multivariate datasets (*55*, *56*). PCA using the singular value decomposition approach of the mean-centered data matrix was used to reduce its dimensionality from *E* to *N* dimensions by expressing the dataset using only the first *N* PCs. The first *N* PCs explain most of the data’s variance, and in this case, six pieces were selected (see Supplementary Figures). The distance between data points in the *N*-dimensional component space (score plot) is a direct measure of the similarity of the XRF spectra and can thus be used to cluster pixels according to their (Euclidean) distances from the cluster centers (centroid linkage method, *k*-means clustering). Pixels with similar spectra are effectively clustered via this method into *k* groups, resulting in an image segmentation. To achieve a finer image segmentation, *k* = 13 clusters were used. After the *k*-means clustering, the result was improved by a GMM using EM for clustering (maximum of 1000 iterations).

#### MA-XRPD imaging

The macro-XRPD imaging experiments on the painting *Still Life with Flowers and a Watch* were carried out using an in-house built mobile MA-XRF/XRPD scanning instrument from the AXIS Research Group (University of Antwerp), operating in reflection mode. A monochromatic Cu-K_α_ (8.04 keV) x-ray source (Incoatec GmbH, Germany) was used with a photon flux of 2.9 × 10^8^ photons/s, a focal diameter of ca. 140 μm, a focal distance of ca. 20 cm, and a divergence of 2.4 mrad. An incident angle of 10° was chosen between the primary x-ray beam and the painting’s surface, leading to a beam with an elliptical footprint of approximately 1 mm by 0.2 mm. Opposite the x-ray source a, PILATUS 200K detector (Dectris Ltd., Switzerland) was positioned at an angle of 40° with the painting surface to record the diffraction patterns. To ensure an equal distance between the x-ray source and the painting for every point of the scan, a laser distance sensor (Baumer Hold., Switzerland) was used to automatically correct the position of the setup for topographical variations. The XRPD scanning instrument is also equipped with a Vortex silicon drift detector (Hitachi, Japan) that allows for simultaneously acquiring XRF data. The above-mentioned equipment is fastened to a motorized platform (motor stages from Newport Corp., USA) that allows moving in the *XY* plane. The painting itself was positioned on a motorized stage that allowed movement in the vertical (*Z*) direction. The area of the yellow rose (97.5 mm by 109.5 mm) was scanned with an exposure time of 10 s per pixel and a step size of 1.5 mm by 1.5 mm.

The recorded XRPD data were processed using the in-house developed software package XRDUA from the AXIS research group (University of Antwerp) (*49*, *57*) The obtained 2D distributions shown in this article are based on the intensity scaling factor obtained from the fitting procedure.

#### Visible–to–near-infrared RIS

VNIR (400 to 1000 nm) diffused reflectance image cubes were acquired using a high sensitivity hyperspectral camera (Surface Optics Corp, 710E model), equipped with a transmission grating-prism spectrometer combined with a backside illuminated EMCCD detector. The imaging camera has a spectral sampling of 2.5 nm, for a total of 260 channels, and produces image cubes with 1024 by 1024 spatial pixels. The spatial resolution at the painting was 0.168 mm, corresponding to 172-mm field of view, and the integration time used was 100 ms. Two light sources Solux 4700K, 50-W lamps (coated to minimize UV and IR light) were used to collect the reflected signal. A step/stare collection approach was used to collect VNIR image cubes of the entire surface of the painting to allow generating a complete image cube. To do this, the camera and lights remained stationary, the painting was moved left-right and up-down on an easel, and a total of 20 cubes (1024 × 1024 pixels) were acquired to have the final VNIR reflectance data cube. The conversion to apparent reflectance was done using a standard protocol, namely flat fielding. A dark image cube (no light allowed into the camera) was collected along with an image cube of the illumination light reflected off a white diffuse reflectance standard (99% reflector, Labsphere, Inc) that was placed in the plane of the painting. The apparent reflectance image cubes were calculated by dividing each raw cube collected of the painting after subtraction of the dark image cube by the image cube of the dark-subtracted diffuse reflectance standard. The 20 cubes were then stitched and registered to a visible image (*58*). The reflectance maps of the endmembers were made using the spectral angle mapper algorithm in the ENVI software (Harris Corp.).

#### Five-micrometer resolution photography

The detail of the yellow rose (10 cm by 10 cm) was photographed in 5-μm resolution with a Hasselblad H6D-400c MS camera by photographer Carola van Wijk. Six separate images were collected with an 11,600 × 18700 pixel dimension at 300 dpi horizontal and vertical resolution. The separate images were color-calibrated using an ICC profile (Argyll) that was custom-made by photographing a ColorChecker Digital SG (X-rite) color card at the same 5-μm resolution. The color-calibrated images were then digitally assembled into a single image in Photoshop.

#### High-resolution 3D digital microscopy

The paint surface of the yellow rose was studied and photographed using a Hirox RH-2000 3D digital microscope on a motorized XY stage, equipped with a MXB-2500REZ lens. The microscope can achieve spatial sampling from 4.3 μm per pixel in low range (×35 magnification), 1.13 μm per pixel in mid-range (×140 magnification), and 0.45 μm per pixel in high range (×350 magnification). For the images in this paper, images were taken in mid-range. A white balance was made with a color checker white balance target of the color checker passport from X-rite. The images were photographed under the same light conditions and exposure time.

## Supplementary Materials

### This PDF file includes:

Figs. S1 to S6 References
