# Supplementary material for: Reviving degraded colors of yellow flowers in 17th century still life paintings with macro- and microscale chemical imaging
Source: Sci Adv. 2022 Jun 8;8(23):eabn6344. doi: 10.1126/sciadv.abn6344 (PMC9176749; doi:10.1126/sciadv.abn6344)
Supplement: Supplementary file 1 — Figs. S1 to S6 References [file sciadv.abn6344_sm.pdf]

Science Advances
AAAS

Supplementary Materials for

Reviving degraded colors of yellow flowers in 17th century still life paintings with macro- and microscale chemical imaging

Nouchka De Keyser *et al.*

Corresponding author: Nouchka De Keyser, n.de.keyser@rijksmuseum.nl

Sci. Adv. **8**, eabn6344 (2022)
DOI: 10.1126/sciadv.abn6344

This PDF file includes:

Figs. S1 to S6
References

Fig. S1.

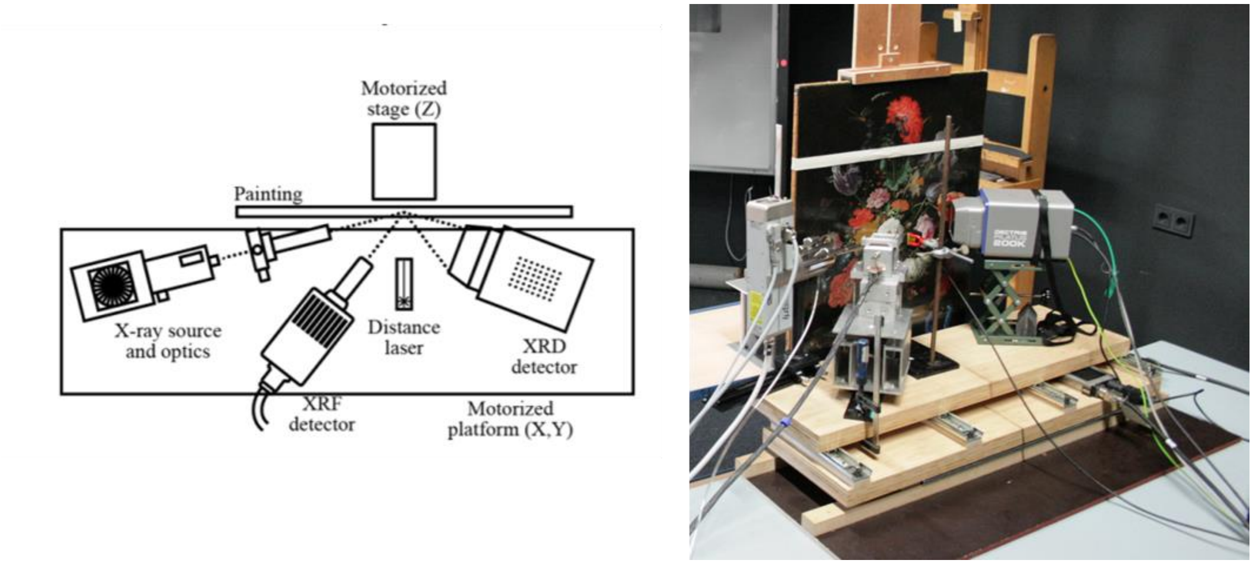

Fig. S1. Overview of the MA-XRPD scanning instrument. Schematics and corresponding visual photograph of the MA-XRPD scanner in reflection mode. Photo Credit: (left) Steven De Meyer, Frederik Vanmeert and (right) Nouchka De Keyser

Fig. S2.

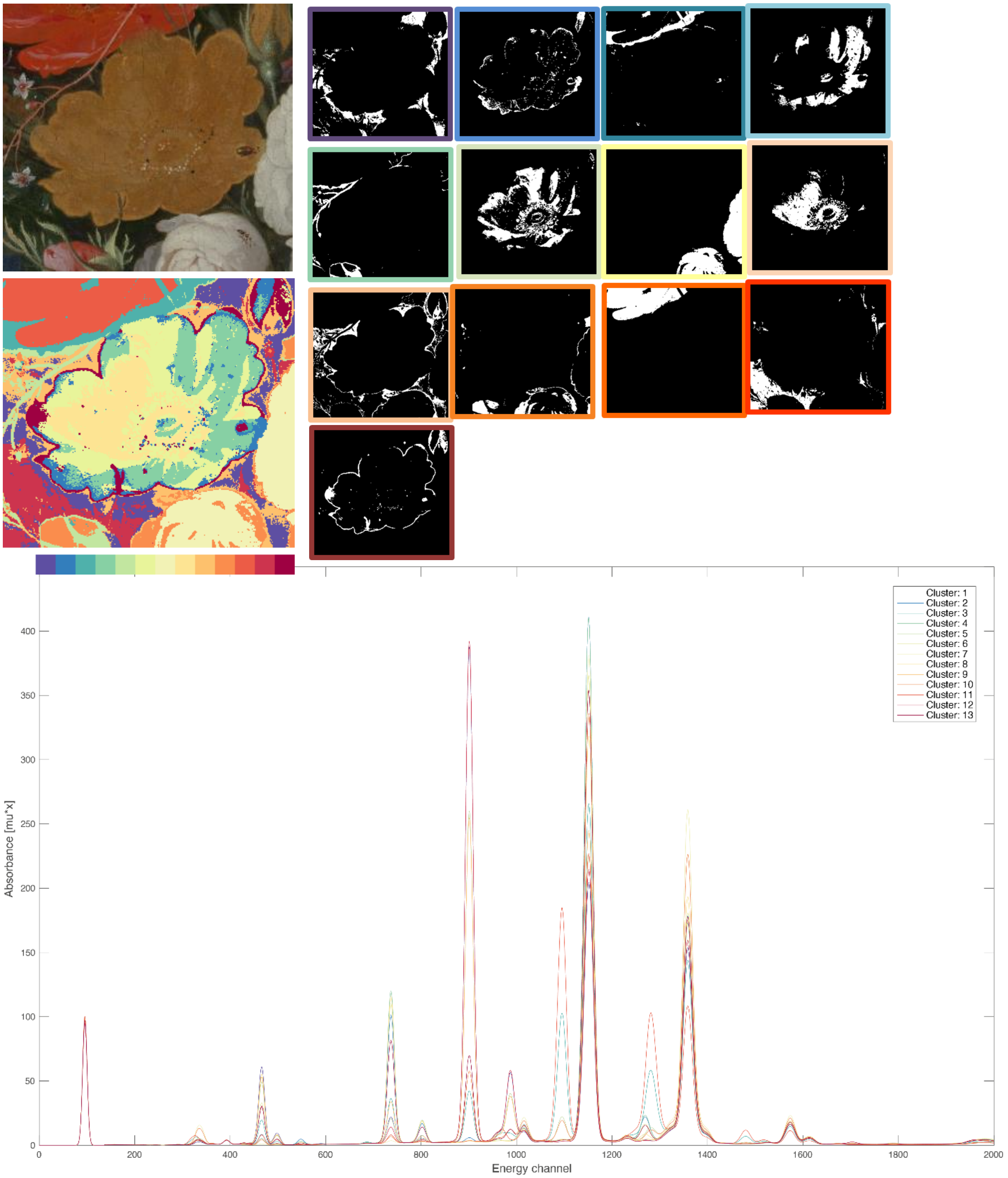

Fig. S2. Results of the PCA k-means clustering (XANES-Wizard). Composite image of the 13 clusters obtained with Principal Component Analysis and subsequent k-means clustering, improved by a Gaussian Mixture Model (GMM) using Expectation Maximization (EM) for clustering (max 1000 iterations) of the MA-XRF dataset.

Fig. S3.

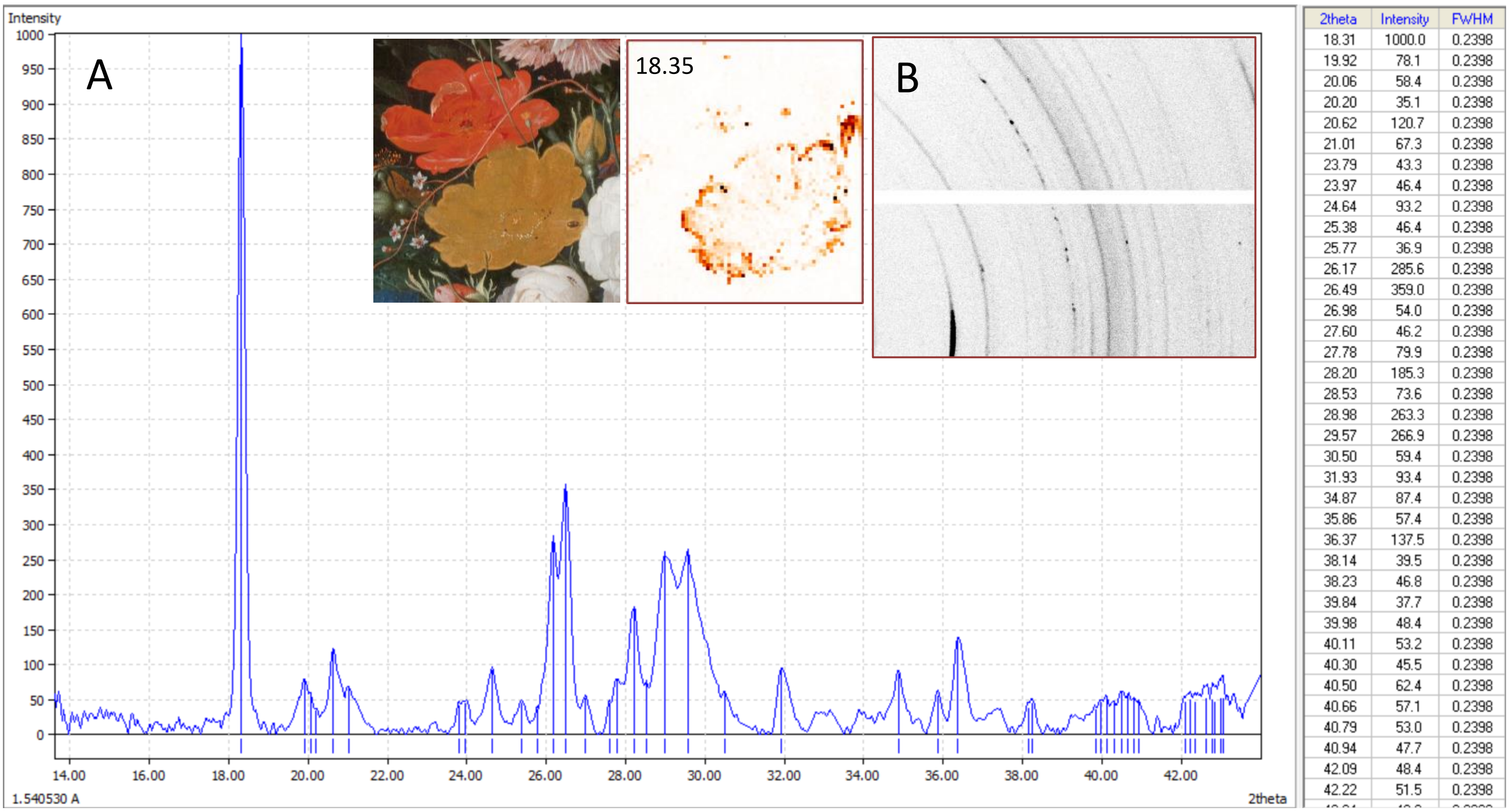

2theta	Intensity	FWHM
18.31	1000.0	0.2398
19.92	78.1	0.2398
20.06	58.4	0.2398
20.20	35.1	0.2398
20.62	120.7	0.2398
21.01	67.3	0.2398
23.79	43.3	0.2398
23.97	46.4	0.2398
24.64	93.2	0.2398
25.38	46.4	0.2398
25.77	36.9	0.2398
26.17	285.6	0.2398
26.49	359.0	0.2398
26.98	54.0	0.2398
27.60	46.2	0.2398
27.78	79.9	0.2398
28.20	185.3	0.2398
28.53	73.6	0.2398
28.98	263.3	0.2398
29.57	266.9	0.2398
30.50	59.4	0.2398
31.93	93.4	0.2398
34.87	87.4	0.2398
35.86	57.4	0.2398
36.37	137.5	0.2398
38.14	39.5	0.2398
38.23	46.8	0.2398
39.84	37.7	0.2398
39.98	48.4	0.2398
40.11	53.2	0.2398
40.30	45.5	0.2398
40.50	62.4	0.2398
40.66	57.1	0.2398
40.79	53.0	0.2398
40.94	47.7	0.2398
42.09	48.4	0.2398
42.22	51.5	0.2398
[illegible]	[illegible]	[illegible]

Fig. S3. **XRPD data orpiment**. (A) 1D diffractogram of a pixel from the border of the flower with orpiment like particles. (B) 2D XRD pattern showing preferential orientation for the high intensity peak around 18.35.

The identification of orpiment was complicated by a strong preferred orientation observed in the 2D XRPD patterns. The preferred orientation is an intrinsic property of certain minerals to orient themselves in certain directions according to a preferred crystallographic plane.(*59*) Ideally, all crystallites are assumed to be randomly oriented for homogenous debye markers, however, when preferred orientations are observed, anisotropic signals are obtained and some reflection may completely disappear.(*5*) This complicates the identification of the pigment as this can lead to the change in relative intensities of the reflections from other crystallographic planes and increasing intensities of peaks corresponding to the preferred oriented cleavage planes.(*59*) This was also the case here, where the most intense peak of orpiment was found at 18.35 (corresponding to the literature and XRD reference data (COD, crystallography open database), but the intensities at the other angular positions deviated from the reference data. With our MA-XRPD set-up (Fig. S1) we also capture a limited azimuthal range with the XRPD detector, which means that there is a great chance that signals showing preferential orientation and thus covering a limited azimuthal range will be lost. Despite the incomplete characterization with XRPD, its distribution corresponded strongly with the defined area I, around the outlines and in certain details of the yellow rose, which is characterized by yellow micaceous, foliated pigment particles, that were identified by Raman as natural orpiment in the cross-section.

Fig. S4.

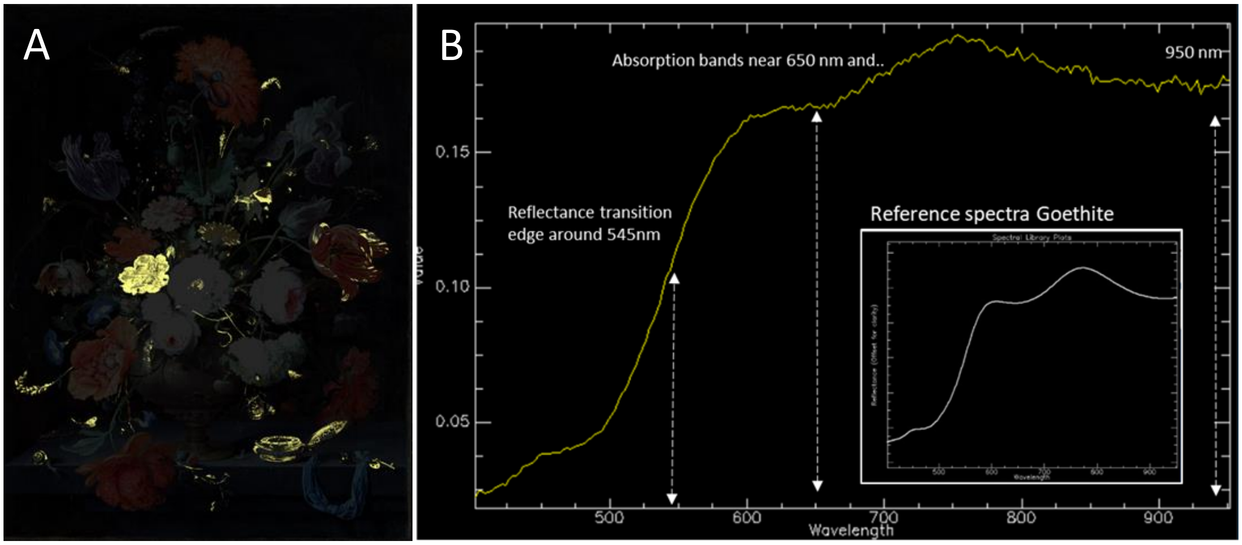

Fig. S4. **Identification and mapping of goethite with Visible-to-near-infrared reflectance imaging spectroscopy of Mignon's *Still life with flowers and a Watch*.** **(A)** Map obtained with the portion of the spectral endmember **(B)** showing characteristic features of Fe Hydroxide know as goethite, contained in the yellow ochre pigment.

Fig. S5.

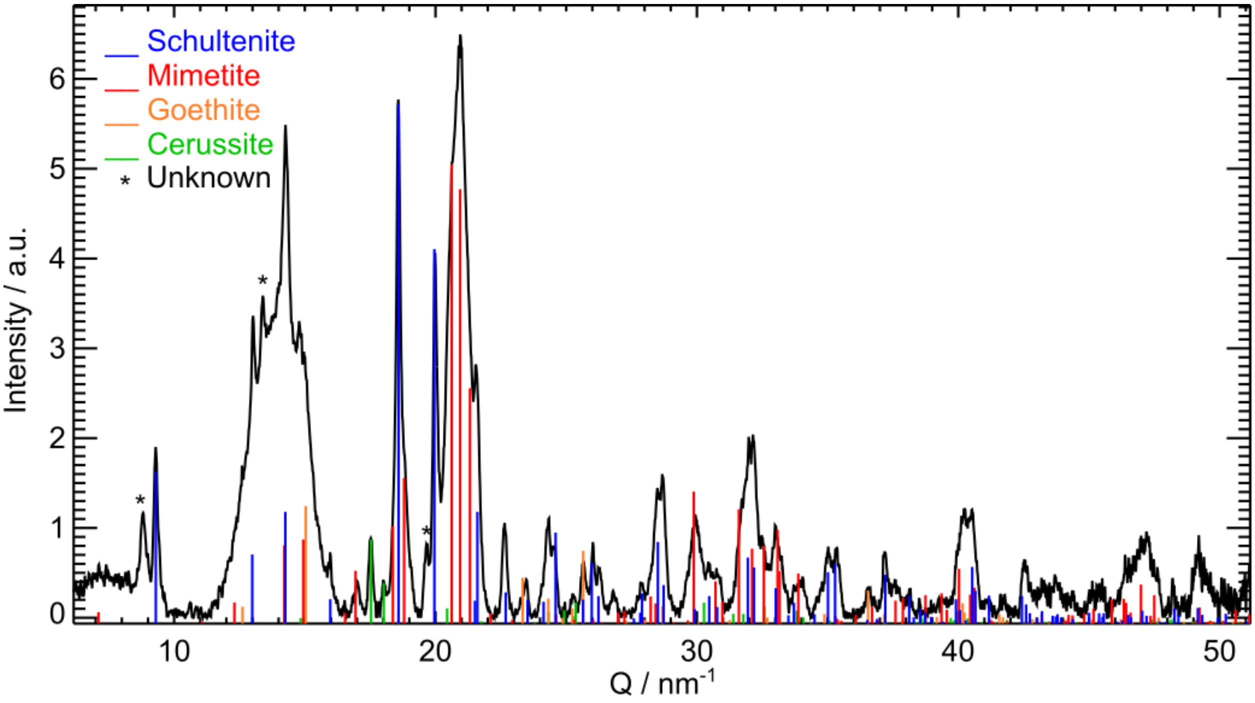

Fig. S5. **Identification of secondary formed arsenic products schultenite and mimetite with SR-µ-XRPD.** Diffractogram obtained after median azimuthal integration of an averaged 2D diffraction image (25 pixels) collected with SR-µ-XRPD.

Fig. S6.

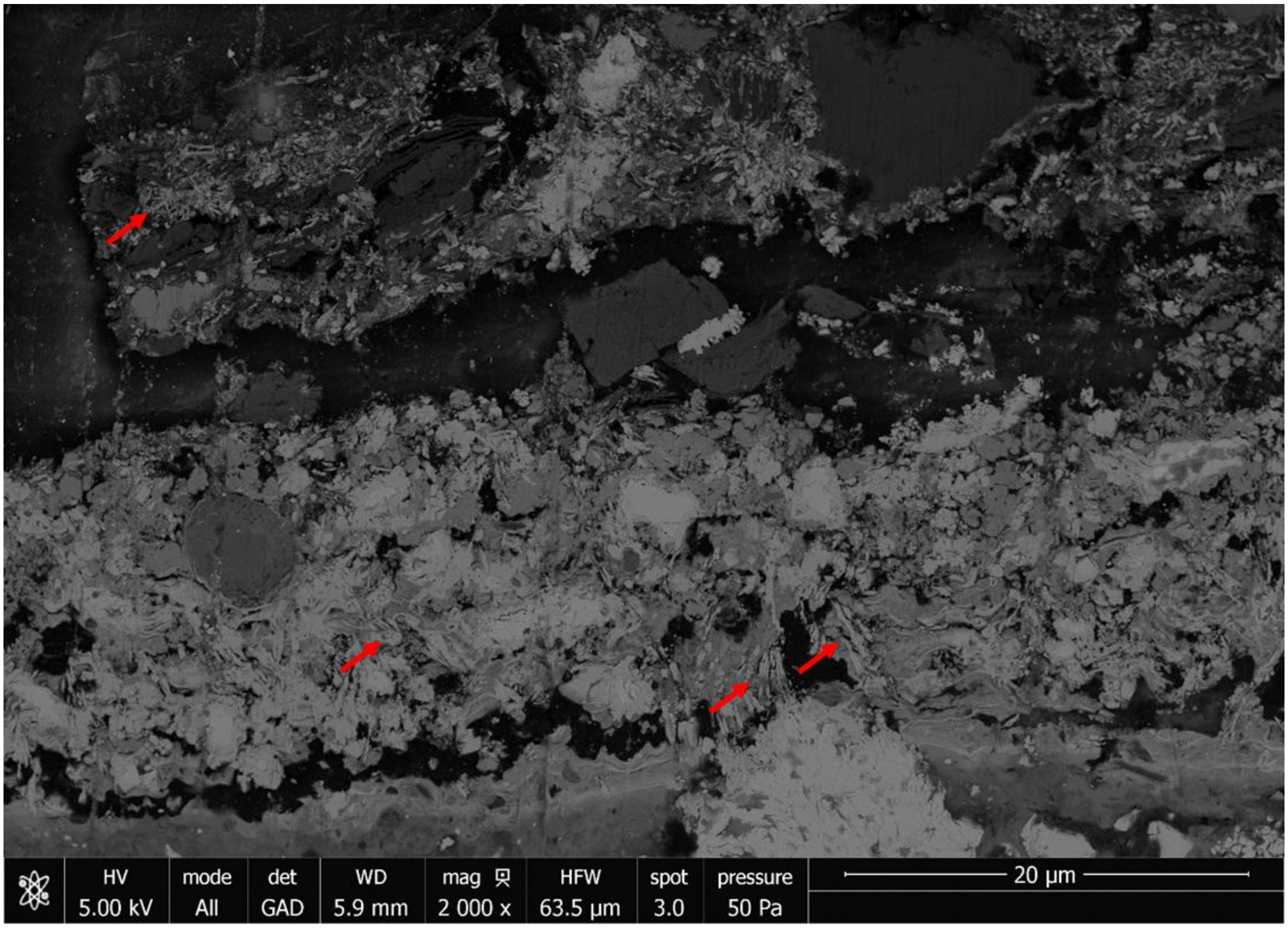

Fig. S6. **Backscattered electron image of cross-section R50/1.** The backscattered electron image (BEI) of cross-section R50/1 shows sharp needle like particles (red arrows), rich in arsenic and lead, visible throughout layer 2-4.

REFERENCES AND NOTES

1. L. Baij, thesis, Universiteit van Amsterdam, Amsterdam (2020).

2. C. Miliani, L. Monico, M. J. Melo, S. Fantacci, E. M. Angelin, A. Romani, K. Janssens, Photochemistry of artists' dyes and pigments: Towards better understanding and prevention of colour change in works of art. *Angew. Chem. Int. Ed.* **57**, 7324–7334 (2018).

3. A. van Loon, P. Noble, D. de Man, M. Alfeld, T. Callewaert, G. Van der Snickt, K. Janssens, J. Dik, The role of smalt in complex pigment mixtures in Rembrandt's Homer 1663: Combining MA-XRF imaging, microanalysis, paint reconstructions and OCT. *Herit. Sci.* **8**, 90 (2020).

4. K. Schnetz, A. A. Gambardella, R. van Elsas, J. Rosier, E. E. Steenwinkel, A. Wallert, P. D. Iedema, K. Keune, Evidence for the catalytic properties of ultramarine pigment. *J. Cult. Herit.* **45**, 25–32 (2020).

5. F. Vanmeert, E. Hendriks, G. Van der Snickt, L. Monico, J. Dik, K. Janssens, Chemical mapping by macroscopic x-ray powder diffraction (MA-XRPD) of Van Gogh's *Sunflowers*: Identification of areas with higher degradation risk. *Angew. Chem. Int. Ed.* **57**, 7418–7422 (2018).

6. L. Monico, L. Cartechini, F. Rosi, A. Chieli, C. Grazia, S. De Meyer, G. Nuyts, F. Vanmeert, K. Janssens, M. Cotte, W. De Nolf, G. Falkenberg, I. C. A. Sandu, E. S. Tveit, J. Mass, R. P. de Freitas, A. Romani, C. Miliani, Probing the chemistry of CdS paints in *The Scream* by in situ noninvasive spectroscopies and synchrotron radiation x-ray techniques. *Sci. Adv.* **6**, eaay3514 (2020).

7. Research project REVIGO, "Original colours of Van Gogh's paintings"; https://assets.vangoghmuseum.nl/a41553fa-025e-4a8d-9e49-132f267652aa?c=fdacfed0591e798c2daa6b72e18b4abc00da7f192ad37b8aca211e2a0c6ace30.

8. G. Van der Snickt, K. A. Dooley, J. Sanyova, H. Dubois, J. K. Delaney, E. M. Gifford, S. Legrand, N. Laquiere, K. Janssens, Dual mode standoff imaging spectroscopy documents the painting process of the Lamb of God in the *Ghent Altarpiece* by J. and H. Van Eyck. *Sci. Adv.* **6**, eabb3379 (2020).

9. S. Legrand, M. Alfeld, F. Vanmeert, W. De Nolf, K. Janssens, Macroscopic Fourier transform infrared scanning in reflection mode (MA-rFTIR), a new tool for chemical imaging of cultural heritage artefacts in the mid-infrared range. *Analyst* **139**, 2489–2498 (2014).

10. K. A. Dooley, D. M. Conover, L. D. Glinsman, J. K. Delaney, Complementary standoff chemical imaging to map and identify artist materials in an early Italian renaissance panel painting. *Angew. Chem.* **126**, 13995–13999 (2014).

11. F. Vanmeert, thesis, Universiteit Antwerpen, Antwerpen (2019).

12. S. De Meyer, F. Vanmeert, R. Vertongen, A. van Loon, V. Gonzalez, J. Delaney, K. Dooley, J. Dik, G. Van der Snickt, A. Vandivere, K. Janssens, Macroscopic x-ray powder diffraction imaging reveals Vermeer's discriminating use of lead white pigments in *Girl with a Pearl Earring*. *Sci. Adv.* **5**, eaax1975 (2019).

13. J. Simoen, S. De Meyer, F. Vanmeert, N. de Keyser, E. Avranovich, G. Van der Snickt, A. Van Loon, K. Keune, K. Janssens, Combined micro- and macro scale x-ray powder diffraction mapping of degraded Orpiment paint in a 17th century still life painting by Martinus Nellius. *Herit. Sci.* **7**, 83 (2019).

14. K. Keune, J. Mass, A. Mehta, J. Church, F. Meirer, Analytical imaging studies of the migration of degraded orpiment, realgar, and emerald green pigments in historic paintings and related conservation issues. *Heri. Sci.* **4**, 10 (2016).

15. K. Keune, J. Mass, F. Meirer, C. Pottasch, A. van Loon, A. Hull, J. Church, E. Pouyet, M. Cotte, A. Mehta, Tracking the transformation and transport of arsenic sulfide pigments in paints: Synchrotron-based x-ray micro-analyses. *J. Anal. At. Spectrom* **30**, 813–827 (2015).

16. C. Pottasch, K. Mench, in *Historical Technology, Materials and Conservation: SEM and Microanalysis* (Archetype Publications, 2012), pp. 100–106.

17. H. Cutts, L. Harrison, C. Higgitt, The image revealed: Study and conservation of a mid-nineteenth century Ethiopian church painting. *Brit. Mus. Tech. Res. Bull.* **4**, 1–17 (2010).

18. S. Van Dorst, Daniël Seghers, phoenix of flower-painters. *Hamilton Kerr Inst. Bull.* **6**, 29–44 (2016).

19. A. Wallert, Orpiment and realgar. *Maltechnik Restauro*, 45–57 (1984).

20. D. L. Douglass, C. Shing, G. Wang, The light-induced alteration of realgar to pararealgar. *Am. Mineral.* **77**, 1266–1274 (1992).

21. F. Vanmeert, N. de Keyser, A. van Loon, L. Klaassen, P. Noble, K. Janssens, Transmission and reflection mode macroscopic x-ray powder diffraction imaging for the noninvasive visualization of paint degradation in still life paintings by Jan Davidsz. de Heem. *Anal. Chem.* **91**, 7153–7161 (2019).

22. M. Vermeulen, G. Nuyts, J. Sanyova, A. Vila, D. Buti, J.-P. Suuronen, K. Janssens, Visualization of As(III) and As(V) distributions in degraded paint micro-samples from Baroque- and Rococo-era paintings. *J. Anal. At. Spectrom* **31**, 1913–1921 (2016).

23. A. Wallert, J. Dik, The scientific examination of a seventeenth-century masterpiece. *Zeitschrift für Kunsttechnologie und Konservierung: ZKK.* **21**, 38–51 (2007).

24. A. Wallert, A. van den Berg, *Still Lifes: Techniques and Style: an Examination of Paintings from the Rijksmuseum* (Rijksmuseum Waanders, 1999).

25. A. H. Christensen, E. Hermens, H. Tempest, in *Painting Techniques: History, Materials and Studio Practice*, A. Wallert, Ed. (Rijksmuseum, 2013), pp. 109–114.

26. N. De Keyser, G. Van der Snickt, A. Van Loon, S. Legrand, A. Wallert, K. Janssens, Jan Davidsz. de Heem (1606–1684): A technical examination of fruit and flower still lifes

combining MA-XRF scanning, cross-section analysis and technical historical sources. *Heri. Sci.* **5**, 38 (2017).

27. S. De Meyer, F. Vanmeert, R. Vertongen, A. van Loon, V. Gonzalez, G. van der Snickt, A. Vandivere, K. Janssens, Imaging secondary reaction products at the surface of Vermeer's Girl with the Pearl Earring by means of macroscopic x-ray powder diffraction scanning. *Heri. Sci.* **7**, 67 (2019).

28. M. C. F. Magalhães, M. C. M. Silva, Stability of lead(II) arsenates. *Monatshefte fuer Chemie/Chemical Monthly* **134**, 735–743 (2003).

29. G. Van der Snickt, H. Dubois, J. Sanyova, S. Legrand, A. Coudray, C. Glaude, M. Postec, P. Van Espen, K. Janssens, Large-area elemental imaging reveals Van Eyck's original paint layers on the Ghent Altarpiece (1432), rescoping its conservation treatment. *Angew. Chem. Int. Ed. Engl.* **56**, 4797–4801 (2017).

30. K. A. Dooley, E. M. Gifford, A. van Loon, P. Noble, J. G. Zeibel, D. M. Conover, M. Alfeld, G. Van der Snickt, S. Legrand, K. Janssens, J. Dik, J. K. Delaney, Separating two painting campaigns in Saul and David, attributed to Rembrandt, using macroscale reflectance and XRF imaging spectroscopies and microscale paint analysis. *Heri. Sci.* **6**, 46 (2018).

31. M. Aceto, A. Agostino, G. Fenoglio, A. Idone, M. Gulmini, M. Picollo, P. Ricciardi, J. K. Delaney, Characterisation of colourants on illuminated manuscripts by portable fibre optic UV-visible-NIR reflectance spectrophotometry. *Anal. Methods* **6**, 1488–1500 (2014).

32. M. J. N. Stols-Witlox, "Historical recipes for preparatory layers for oil paintings in manuals, manuscripts and handbooks in North West Europe, 1550–1900: analysis and reconstructions," thesis, Universiteit van Amsterdam, Amsterdam (2014).

33. M. Vermeulen, thesis, Universiteit Antwerpen, Antwerpen (2017).

34. J. Kirby, M. Spring, C. Higgitt, The technology of Red Lake pigment manufacture: Study of the dyestuff substrate. *Natl. Gallery Tech. Bull.* **26**, 71–87 (2005).

35. D. Saunders, J. Kirby, Light-induced colour changes in red and yellow lake pigments. *Natl. Gallery Tech. Bull.* **15**, 79–97 (1994).

36. E. Hermens, A. Wallert, in *Looking Through Paintings: The Study of Painting Techniques and Materials in Support of Art Historical Research*, E. Hermens, Ed. (De Prom, Baarn; Archetype, London, 1998), pp. 269–295.

37. A. van Loon, thesis, Universiteit van Amsterdam (2008).

38. R. D. Harley, *Artists Pigments c. 1600–1835: A Study in English Documentary Sources* (Butterworth Scientific, 1982).

39. G. P. Lomazzo, Trattato dell'arte de la pintura, Libro Terzo: Del Colore, Cap VI (1584).

40. M. Spring, Colourless powdered glass as an additive in fifteenth-and sixteenth-century European paintings. *Natl. Gallery Tech. Bull.* **33**, 4–26 (2012).

41. C. Cennini, G. Tambroni, M. P. (Mary P. Merrifield), *A Treatise on Painting, written by Cennino Cennini in the year 1437* (London, Lumley, reprint by BiblioBazaar, 1844).

42. M. Smith, *The Art of Painting According to the Theory and Practise of the Best Italian, French and Germane Masters* (1692).

43. P. Y. Velasco, A, El museo pictoricó y la escala óptica (1724), vol. 2.

44. T. T. de Mayerne, Pictoria, sculptoria, tinctoria et quae subalkternarum Artium spectantia (1620).

45. Beurs, Wilhelmus, *De groote waereld in't kleen geschildert, of Schilderagtig tafereel van's weerelds schilderyen, kortelijk vervat in ses boeken: verklarende de hooftverwen, haare verscheide mengelingen in oly, en der zelver gebruik. Omtrent de meeste vertoningen van de zigtbare natuire. Leersaamelijk den liefhebbers en leerlingen der ed. schilderkonst medegedeelt* (Johannes en Gillis Janssonius van Waesberge, T'Amsterdam, 1692).

46. K. Lutzenberger, H. Stege, C. Tilenschi, A note on glass and silica in oil paintings from the 15th to the 17th century. *J. Cult. Herit.* **11**, 365–372 (2010).

47. K. Groen, *Paintings in the Laboratory: Scientific Examination for Art History and Conservation* (Archetype Publications, 2014).

48. C. G. Schroer, P. Boye, J. M. Feldkamp, J. Patommel, D. Samberg, A. Schropp, A. Schwab, S. Stephan, G. Falkenberg, G. Wellenreuther, N. Reimers, Hard x-ray nanoprobe at beamline P06 at PETRA III. *Nucl. Instrum. Methods Phys. Res. A* **616**, 93–97 (2010).

49. W. De Nolf, F. Vanmeert, K. Janssens, XRDUA: Crystalline phase distribution maps by two-dimensional scanning and tomographic (micro) x-ray powder diffraction. *J. Appl. Cryst.* **47**, 1107–1117 (2014).

50. M. Alfeld, J. V. Pedroso, M. van E. Hommes, G. V. der Snickt, G. Tauber, J. Blaas, M. Haschke, K. Erler, J. Dik, K. Janssens, A mobile instrument for in situ scanning macro-XRF investigation of historical paintings. *J. Anal. At. Spectrom* **28**, 760–767 (2013).

51. M. Alfeld, K. Janssens, Strategies for processing mega-pixel x-ray fluorescence hyperspectral data: A case study on a version of Caravaggio's painting Supper at Emmaus. *J. Anal. At. Spectrom* **30**, 777–789 (2015).

52. V. A. Solé, E. Papillon, M. Cotte, Ph. Walter, J. Susini, A multiplatform code for the analysis of energy-dispersive x-ray fluorescence spectra. *Spectrochim. Acta B At. Spectrosc.* **62**, 63–68 (2007).

53. Y. Liu, F. Meirer, P. A. Williams, J. Wang, J. C. Andrews, P. Pianetta, TXM-Wizard: A program for advanced data collection and evaluation in full-field transmission x-ray microscopy. *J. Synchrotron. Rad.* **19**, 281–287 (2012).

54. F. Meirer, Y. Liu, E. Pouyet, B. Fayard, M. Cotte, C. Sanchez, J. C. Andrews, A. Mehta, P. Sciau, Full-field XANES analysis of Roman ceramics to estimate firing conditions—A novel probe to study hierarchical heterogeneous materials. *J. Anal. At. Spectrom* **28**, 1870 (2013).

55. S. Vogt, J. Maser, C. Jacobsen, in *Journal de Physique IV (Proceedings)* (EDP sciences, 2003), vol. 104, pp. 617–622.

56. I. Tsodoulos, C. Papachristodoulou, K. Stamoulis, K. Ioannides, S. Pavlides, R. Caputo, A. Chatzipetros, I. Koukouvelas, E. Kremastas, in *EGU General Assembly Conference Abstracts* (2013).

57. W. De Nolf, thesis, University of Antwerp, Antwerp (2013).

58. D. M. Conover, J. K. Delaney, M. H. Loew, Automatic registration and mosaicking of technical images of Old Master paintings. *Appl. Phys. A Mater. Sci. Process.* **119**, 1567–1575 (2015).

59. A. L. da Silva, A. H. de Oliveira, M. L. S. Fernandes, Influence of preferred orientation of minerals in the mineralogical identification process by x-ray diffraction (International Nuclear Atlantic Conference-INAC 2011 Proceedings, Associacao Brasileira de Energia Nuclear, 2011).
